# From evidence to implementation: a policy and health systems roadmap for cervical cancer elimination in India

**DOI:** 10.3389/fpubh.2026.1906243

**Published:** 2026-09-18

**Authors:** Latha Balasubramani, Purnima Madhivanan, Meenu Anand, Mridu Gupta, Priya Ganesh Kumar, Abhishek Shankar, Shobha S. Krishnan

**Affiliations:** 1 Department of Oncology, GKNM Hospital, Coimbatore, India; 2 Public Health Research Institute of India, Mysuru, Karnataka, India; 3 Global Cancer Prevention and Screening, American Cancer Society, Atlanta, GA, United States; 4 Cancer Awareness, Prevention and Early Detection (CAPED), Gurugram, Haryana, India; 5 Preventive Oncology, Sainiwas Healthcare, Mumbai, India; 6 Department of Radiation Oncology, All India Institute of Medical Sciences (AIIMS), New Delhi, India; 7 Global Initiative Against HPV and Cervical Cancer (GIAHC), Durham, NC, United States

**Keywords:** cervical cancer elimination, health systems, HPV vaccination, implementation science, India, policy synthesis, screening

## Abstract

**Background:**

Cervical cancer is the first cancer that can be eliminated, yet it remains a leading cause of cancer-related mortality among women in low- and middle-income countries. India accounts for a substantial share of the global burden, making its trajectory central to global elimination efforts. Although effective tools—including HPV vaccination, HPV-based screening, and treatment of precancerous lesions—are well established, progress has been uneven. The central challenge is no longer scientific discovery but large-scale implementation, building on India's demonstrated capacity for nationwide public health delivery through polio eradication, HIV/AIDS control, and COVID-19 vaccination.

**Methods:**

We conducted a conference-informed policy and implementation synthesis drawing on expert presentations from the International Papillomavirus Society (IPVS) 2025 “The World Could End Cervical Cancer If it Tried: spotlight on India” session, complemented by peer-reviewed literature and policy documents. A rapid thematic synthesis approach was used to identify and organize implementation priorities across the prevention and care continuum.

**Findings:**

Five interrelated priorities emerged. First, demand generation must move beyond awareness to trust-driven uptake through credible messengers and clear service pathways. Second, strengthening provider capacity is essential to improve vaccine confidence and recommendation practices across the clinical workforce. Third, screening programmes must be redesigned around completion of the care cascade rather than participation alone, with integrated pathways for triage and treatment. Fourth, community engagement should be embedded within service delivery and navigation systems to translate trust into sustained participation and follow-through. Fifth, governance, financing, and digital infrastructure—including national registries and interoperable data systems—are critical to enable coordinated, accountable scale-up. Across all domains, trust functions as a central cross-cutting mechanism linking awareness to uptake and continuity of care.

**Interpretation:**

Cervical cancer elimination in India is achievable but requires a shift from fragmented initiatives to coordinated national implementation. Bridging the gap between evidence and delivery will depend on aligning communication, workforce capacity, service integration, community engagement, and system-level governance with urgency and accountability. India's success will not only determine national outcomes but will be pivotal to achieving global cervical cancer elimination within this generation.

## Introduction

Cervical cancer remains a leading cause of cancer-related morbidity and mortality among women in low- and middle-income countries, with India accounting for approximately one-fifth to one-quarter of the global burden (1). An estimated 511.4 million women aged 15 years and older are at risk of developing cervical cancer in India (2). The WHO Global Strategy identifies girls aged 9–14 years as the primary target group for HPV vaccination, with the goal of fully vaccinating 90% of girls by age 15 years by 2030 (3). Late-stage diagnosis remains common, resulting in poorer outcomes and higher treatment costs.

In 2020, the World Health Organization (WHO) launched the Global Strategy to Accelerate the Elimination of Cervical Cancer as a Public Health Problem, defining elimination as fewer than four cases per 100,000 women and establishing the 90–70–90 targets for vaccination, screening, and treatment by 2030 (3). Without intervention, an estimated 5.8 million women in India are projected to die from cervical cancer between 2020 and 2070, rising to 13.3 million deaths by 2120. Successful implementation of the triple-pillar strategy could save approximately 3.6 million lives over 50 years and 10.7 million lives over 100 years (4, 5).

India remains far from achieving these targets. Despite recent announcements of a national HPV vaccination campaign, vaccine coverage is limited, screening rates are low, and access to timely diagnosis and treatment is uneven across regions (6). Modeling studies in LMICs indicate that without accelerated scale-up of vaccination and HPV-based screening, elimination will be delayed, whereas integrated prevention strategies could significantly shorten the timeline to impact (7).

The challenge in India is therefore no longer scientific uncertainty but implementation. Proven tools—including HPV vaccination, HPV DNA-based screening, self-sampling, and treatment of precancerous lesions—already exist. The priority is to scale these interventions through effective demand generation, organized service delivery, continuity across the screening-to-treatment cascade, strengthened governance, and sustainable financing in a large and heterogeneous health system.

India's successful polio eradication and large-scale vaccination campaigns illustrate that ambitious public health goals can be achieved through sustained political commitment, strong programme governance, adequate financing, and community engagement providing a blueprint for the scale-up of cervical cancer prevention (8). Cervical cancer elimination presents a comparable systems challenge requiring sustained commitment, coordinated delivery, and trust-driven uptake.

## Methods

### Study design

This manuscript presents a conference-informed policy and implementation synthesis on cervical cancer elimination in India. It identifies key delivery, health-system, and policy challenges across vaccination, screening, referral, treatment, and financing, and outlines an actionable roadmap to achieve population-level impact. It applies a narrative, conference-informed synthesis method, integrating expert presentations from the IPVS 2025 session with supporting peer-reviewed and policy literature to construct a stakeholder-informed implementation framework.

### Study setting

The source session was convened as part of the 37th Annual Conference of the International Papillomavirus Society (IPVS 2025), held in October 2025 at Bangkok, Thailand, under the conference theme “Research toward the global elimination of HPV-related diseases and cancers.” The “Spotlight on India” session was organized by the Global Initiative Against HPV and Cervical Cancer (GIAHC) as a special session within this conference, as an in-person, moderated panel format.

### Research team and positionality

The synthesis was led by a gynaecologic oncologist based at a tertiary cancer referral center in India (LB) and a public health physician with long-standing advocacy and programmatic experience in global cervical cancer elimination (SSK), both of whom have prior experience in policy-oriented synthesis. This clinical and public-health positioning informed the selection of implementation-focused themes and priorities. The research team approached the synthesis with the shared premise that effective prevention tools already exist and that delivery, not discovery, was the central barrier to elimination in India.

### Selection of experts for the conference session

Experts were selected by session organizers based on demonstrated experience in HPV vaccination, cervical screening, implementation research, provider education, civil society programming, health communication, and public health policy. They were included for their relevance to on-field implementation, policy engagement, and technical leadership in cervical cancer prevention in India. This purposive selection approach prioritized depth of implementation experience over random or systematic sampling to ensure that the session reflected on – ground expertise.

### Data sources

Conference Materials from the IPVS 2025 Spotlight on India session, including presentations, slide decks, session notes, and moderated discussions were used in the preparation of this manuscript. A desk review of PubMed was conducted for English-language publications from Jan 2015 to June 2026, combining terms for cervical cancer, HPV vaccination, HPV-based screening, self-sampling, implementation science, and health systems, and restricted to India and other low- and middle-income country settings. Policy and programme documents from WHO, the Government of India, and relevant national technical bodies were also included. Titles and abstracts were screened for relevance to the five implementation domains that emerged from the synthesis, and reference lists of included articles and existing policy documents were hand-searched for additional sources. Screening and selection were conducted by a single reviewer using a rapid, narrative-synthesis approach rather than a formal systematic-review protocol. Policy and programme documents.

### Analytical approach

Materials were analyzed using rapid thematic synthesis. Recurring themes identified across presentations and supporting documents were grouped into health-system and implementation domains, refined through narrative integration, and triangulated with external literature and policy guidance. Findings were organized into five implementation areas: (1) demand generation and communication, (2) provider capacity and vaccine confidence, (3) screening and care cascade integration, (4) community-linked service delivery, and (5) governance, digital systems, and financing. Of the literature and policy sources cited to support each domain, three (9–11) relate primarily to demand generation and communication (9–11), two to provider capacity and vaccine confidence (12, 13), eight to screening and care cascade integration (14–21), and 10 (3–5, 22–28) to governance, digital systems, and financing; the section on community-linked service delivery draws primarily on conference discussion rather than published literature), Several sources informed more than one domain.

### Rigor and trustworthiness

Rigor was supported by triangulating conference presentations against peer-reviewed literature and policy documents, so that thematic claims were not based on a single source type, and by maintaining a record linking each thematic claim to its underlying source materials.

## Results

The five domains presented below emerged from thematic synthesis of the conference presentations and supporting literature; they are presented here as an organizing framework for the findings that follow, rather than as an *a priori* structure imposed on the analysis.

## Demand generation and communication: moving from awareness to action

Demand generation is fundamental to the success of HPV vaccination and cervical screening. Evidence shows that communication is most effective when delivered by trusted messengers, framed around outcomes meaningful to families, and linked to clear pathways for accessing services (9). In India, frontline health workers particularly Accredited Social Health Activists (ASHAs) build trust with women and caregivers through consistent household-level engagement and cultural familiarity, proving critical in rural and underserved settings (10).

A key implementation distinction is the difference between awareness generation and service uptake. Communication campaigns, social media content, and digital videos may achieve wide reach, but reach alone does not predict behavior change. Uptake depends on whether awareness is converted into trust, trust into intent, and intent into navigation through available services. This makes communication not an adjunct activity, but a core delivery function within elimination programs.

Evidence from sub-Saharan Africa indicates that communication modalities serve complementary roles in improving HPV vaccination uptake among adolescents. Interpersonal communication by clinicians and community health workers is essential for addressing misinformation and prompting action, whereas mass media and community-based approaches broaden reach and normalize vaccination (9). School-based delivery remains one of the most efficient and equitable vaccination strategies, provided it is supported by consistent provider recommendation, caregiver confidence, and simple operational pathways. Effective programmes combine these approaches rather than rely on a single channel.

To strengthen cancer awareness and prevention, the National Cancer Grid has launched a behavior-driven IEC initiative targeting awareness gaps in rural and underserved communities through culturally sensitive, scientifically based communication tools (11). Such initiatives can improve message consistency across clinical, educational, community, and digital platforms.

At scale, communication strategies should prioritize framing HPV vaccination as cancer prevention, integrating it into routine adolescent health, leveraging trusted messengers, avoiding unnecessary repetition of myths, and linking messages to clear calls to action regarding eligibility, timing, and service delivery. The objective is not only to raise awareness but also to reduce attrition between knowledge and completed vaccination or screening.

## Strengthening provider capacity to improve vaccine confidence and delivery

Health-care providers play a central role in HPV vaccination, as strong provider recommendations significantly increase vaccine initiation and completion. However, providers' knowledge of HPV vaccination remains low, with correspondingly low recommendation rates, and gaps in counseling confidence represent a persistent barrier to uptake (12).

In India, large-scale training initiatives provide a model for strengthening implementation readiness. The Federation of Obstetric and Gynecological Societies of India (FOGSI), in collaboration with the Cancer Foundation of India, implemented a National HPV Vaccination Awareness Project in 2023–2024. In Phase I, 191 master trainers trained 10,800 FOGSI members nationwide, improving knowledge, confidence in vaccine efficacy, and intention to recommend vaccination.

The initiative was later expanded with the Indian medical association (IMA) to include family physicians, pediatricians, and other frontline providers. As of 31 March 2026, 31,500 IMA doctors had completed certification in HPV vaccination. This expansion underscores a key implementation principle: cervical cancer prevention cannot rely on a single speciality but requires engagement across the broader clinical workforce, particularly those interacting with adolescents and families. Inspite of such training initiatives, training coverage, provider knowledge, and recommendation practices vary substantially across India's states and health systems highlighting the need for sustained training initiatives to improve provider capacity.

Pre-service education is equally essential. Evidence indicates that medical students often report feeling unprepared to counsel patients on HPV vaccination, highlighting the need for structured training in pre-service education (13). Integrating cervical cancer prevention into undergraduate and postgraduate curricula would normalize prevention early and strengthen the future workforce.

Taken together, these findings suggest that provider capacity building should be approached as a systems intervention rather than a one-time educational exercise. Effective programmes should include standardized content, practical counseling approaches, cross-specialty reach, periodic refreshers, and integration into formal medical education. Strengthening provider recommendation—one of the strongest predictors of vaccine acceptance and screening uptake—is critical to successful implementation.

## Screening, preinvasive and invasive treatment and care cascade integration: from participation to treatment completion

India's public-sector cervical screening programmes have historically relied on visual inspection with acetic acid (VIA). Although VIA has been widely used for cervical screening in low-resource settings, evidence from multicentre studies demonstrates considerable variability in its sensitivity and specificity, driven by subjectivity and interobserver variation, underscoring its dependence on sustained training and quality assurance (14). HPV-based screening offers substantially higher sensitivity for detecting cervical precancer than VIA, supports longer screening intervals, and enables greater programmatic efficiency at scale—advantages that align with long-term elimination goals and underpin current WHO screening recommendations (15, 16). Despite substantial global evidence supporting the transition to HPV testing, India's operational screening guidelines do not yet include HPV testing as a primary screening strategy. Encouragingly, the Federation of Obstetric and Gynecological Societies of India (FOGSI) is advancing a position statement supporting HPV-based screening, alongside emerging evidence validating an indigenous HPV test that could improve affordability and scalability in India.

Evidence from India supports the feasibility and value of HPV-based approaches, including self-sampling. A cost-utility analysis from Sikkim demonstrated that home-based HPV self-sampling had lower unit costs than VIA and was cost-effective, supporting its potential as a resource-efficient strategy (17). Implementation research highlights operational challenges in transitioning to HPV DNA–based screening such as laboratory logistics, provider readiness, training, and health system adaptation underscoring the importance of integrating new technologies with local health systems and community engagement mechanisms (18, 19).

Screening scale-up is similarly constrained by capacity gaps, including shortages of trained cytotechnologists, colposcopists, and molecular laboratory personnel for HPV testing, as well as uneven distribution of screening infrastructure across urban and rural settings. Strengthening this workforce and infrastructure base would be a pre requisite for expanding screening coverage, similar to how provider capacity underpins vaccination uptake.

The Access Cancer Care India (ACCI) initiative, designed to develop and evaluate co-designed multilevel strategies integrating screening within primary care and helping strengthen referral pathways, provides an emerging model for improving access to the cancer care continuum among rural populations in India. Community-based models incorporating self-sampling are particularly promising for reaching never-screened women and those with a cervix, by reducing barriers related to shame, stigma, privacy, and travel, as demonstrated by programmes such as Program ROSE in Malaysia and other global studies (19–21).

However, the central implementation challenge is not just screening uptake, but completion of the care cascade. A cascade lens is useful because it identifies where women are lost across the continuum: invitation, participation, HPV testing, triage, referral, treatment, and follow-up. Evidence from multiple settings suggests that the greatest attrition often occurs after a positive screen, when women must navigate fragmented referral pathways, travel for treatment, or wait for diagnostic confirmation.

This distinction between screening participation and treatment completion is critical. Increased screening participation is necessary, but insufficient by itself. Programs that improve uptake without ensuring triage and treatment completion may widen the gap between detection and effective care, undermining trust and limiting population-level benefit. For this reason, screening programs should be designed around integrated care pathways that include rapid (or immediate) access to treatment of screen-detected preinvasive disease, referral coordination for diagnostics, LEEP/thermal ablation, invasive cancer treatment access, follow-up mechanisms, and accountability for completion of care.

Implementation priorities include decentralizing treatment where feasible, adopting screen-and-treat or screen–triage–treat approaches, strengthening patient navigation, and establishing digital tracking systems to reduce loss to follow-up. A national screening registry is essential for monitoring performance and guiding corrective action.

## Community-linked service delivery: connecting trust, access, and continuity

Community engagement is essential not only for generating demand but also for ensuring completion across the prevention and care continuum. Programmes led by the Public Health Research Institute of India (PHRII) and the Cancer Awareness, Prevention and Early Detection (CAPED) Trust demonstrate how civil societies can function as implementation partners. These models integrate outreach, awareness, screening facilitation, and referral linkage, particularly in underserved populations, translating community trust into service utilization when appropriate community engagement is connected to care pathways. Evidence from India also shows that culturally grounded, community-based approaches such as storytelling by women who have completed treatment improve uptake and follow-through when linked to accessible services rather than standalone awareness activities.

Another key factor that came up during discussions was the difference between awareness campaigns and navigation support. While campaigns increase awareness, a rapport with the community fosters trust and enables progression from awareness to participation and treatment completion. Frontline workers and community organizations play a critical role in supporting navigation, reducing practical and psychological barriers, and improving follow-up. The initial hesitancy and the fear of the unknown often prevent screen-positive women from accessing necessary health services. If the health system addresses this barrier through compassionate and reassuring patient navigation, these women can become powerful advocates, encouraging others in their communities to seek timely care, thereby setting into motion a ripple effect that strengthens participation and treatment adherence.

Multisectoral platforms such as the Mysore Consortium Against Cervical Cancer highlight the value of coordinated engagement across clinicians, civil society, academia, and media. Their impact should be evaluated using implementation metrics such as screening uptake, referral completion, treatment completion, and retention across the care cascade.

Overall, community engagement should be embedded within structured service delivery pathways. Effective models are likely to be health systems that integrate awareness, service access, navigation, and follow-up supported by robust data systems, rather than treating these as separate components.

## Governance, digital systems, and financing: enabling scale and accountability

The evidence base for cervical cancer prevention and control is strong, but evidence alone does not produce population-level impact. Governance, financing, and digital infrastructure determine whether prevention tools can be delivered equitably, monitored effectively, and sustained over time.

HPV vaccination and HPV-based screening are among the most cost-effective public health interventions available for cervical cancer control, and are endorsed as priority strategies within the WHO Global Strategy for cervical cancer elimination (3, 22). Economic analyses indicate substantial returns on investment, including an estimated USD $10.53 returned for every $1 invested in India under the original elimination strategy (rising to USD $28.29 over a 50-year timeframe), alongside over 3.6 million lives saved by 2070 and more than 10.6 million by 2120 if the 90–70–90 targets are achieved by 2030 (4, 5). Realizing these returns depends on sustained progress toward the 90–70–90 targets and consistent financing and screening quality over multi-decade horizons, so modeled impact is ultimately contingent on real-world implementation fidelity rather than assured by the projections themselves. In addition, HPV accounts for approximately 5% of the global cancer burden, reinforcing its importance as a central prevention strategy.

Recent policy developments in India have created a more favorable enabling environment. The 2024 Union Budget signaled support for HPV vaccination among girls aged 9–14 years (23), while state-level programmes in Sikkim and Punjab demonstrate the feasibility of high-coverage implementation (24). Global momentum, including the 2025 United Nations High-Level Meeting on Non-Communicable Diseases, and national initiatives such as the AIIMS-led summit, further reflect growing alignment between global commitments and national priorities.

However, progress remains fragmented, with many initiatives confined to specific states or districts. Health-system constraints, including high out-of-pocket expenditure and uneven access to cancer care, continue to limit equitable uptake and continuity of services (25). The national HPV vaccination rollout launched in February 2026 represents an important policy milestone, with a single-dose HPV vaccine currently being offered to 14-year-old girls through health facilities. However, vaccine uptake has been uneven, and there has been limited coordinated engagement with parents regarding the importance of HPV vaccination. The programme has also been affected by misinformation campaigns that have undermined public confidence in vaccine safety and efficacy. Ensuring that communities receive accurate, culturally appropriate, and accessible information will be essential to strengthen vaccine confidence and improve uptake, particularly before integration into the Universal Immunization Programme.

A major structural gap is the absence of an organized, population-based national screening programme with robust registry systems. Current efforts remain localized, limiting systematic monitoring of coverage, triage, treatment completion, and outcomes. Global experience shows that sustained reductions in cervical cancer burden require organized screening programmes with registry capacity and quality assurance (26, 27).

Digital systems offer an important opportunity to address these gaps. IARC's CanScreen5 platform provides a framework for monitoring screening programmes and strengthening quality assurance (27). India's U-WIN platform demonstrates scalable digital infrastructure that could support HPV vaccination tracking and follow-up (28). Integrating screening programmes within a national registry architecture aligned with CanScreen5 would be a critical step toward coordinated implementation.

Financing remains a key constraint. Despite the high burden of cancer, global financing remains limited. Emerging mechanisms such as the Global Cancer Financing Platform highlight the need for more structured and sustainable approaches. In India, financing strategies must align with domestic policy ownership, investments in service delivery and workforce capacity, and financial protection for patients.

Overall, cervical cancer elimination is fundamentally a systems challenge. Achieving scale will require coordinated national strategies that align governance, financing, digital systems, and service delivery across the full prevention and care continuum.

## Lessons from the field

The field experience underlying this synthesis points to two central lessons for translating this framework into practice in India. First, community mobilization must sit at the center of both vaccination and screening rather than being treated as a downstream add-on to service availability. Making services available is not sufficient: where communities are hesitant, unaware of where to access care, or distrustful of the service itself, uptake remains low regardless of supply. Intensive, context-appropriate mobilization, using information, education, and communication (IEC) materials in simple, locally understood language and tailored to different stakeholders so that ASHAs, other frontline workers, community leaders, teachers, influencers, parents, and beneficiaries each understand their role is crucial in mobilizing the community. Bringing these voices together builds trust, addresses myths and hesitancy, and enables informed decision-making around vaccination and screening.

Second, screening and vaccination must be built into the existing health system as a single, integrated pathway rather than delivered as standalone activities. Field experience indicates that uptake improves when services are delivered through trusted, existing platforms with a clear pathway from mobilization through vaccination or screening to referral and follow-up. Community awareness, mobilization, counseling, and recruitment can cost nearly as much as the HPV vaccine or HPV DNA test itself, yet approximately half of women who are screen positive may be lost before completing follow-up care; availability alone therefore does not translate into uptake or impact. A comprehensive strategy should invest across interconnected areas: improving knowledge, risk perception, and acceptance of preventive care through behavioral science, social media, culturally appropriate digital tools, peer support, and AI-enabled chatbots. Making vaccination and screening accessible and equitable by reducing the cost of HPV diagnostics; introducing smart technologies, including AI-assisted colposcopy and pathology, to improve efficiency, quality, and scalability while reducing reliance on scarce specialists. It is mandatory that we develop integrated digital health systems that connect outreach, screening, vaccination, diagnosis, treatment, and follow-up through navigation, reminders, telemedicine, and referral tracking.

Implementation research is also needed to address misinformation, hesitancy, and stigma, so that preventive care becomes a routine part of health-seeking behavior rather than an exceptional event. These operational realities were echoed in specific challenges raised from the field. Stigma around a positive screening result means that follow-up cannot always be conducted at the same venue as general screening, since a woman returning to the same site risks being visibly identified as having an abnormal result; follow-up venues therefore may need to be separate and discreet from general screening sites. Supporting screen-positive women through to completed treatment also requires substantial emotional support, hand-holding, and counseling; the models currently used for this are time- and labor-intensive in ways that limit their scalability. More broadly, loss to follow-up among screen-positive women and inequitable access to both vaccination and screening for rural, underserved, and vulnerable populations remain the major implementation challenges confronting India's cervical cancer programme; addressing them will require stronger linkage between screening, diagnostic and treatment pathways than currently exists.

India already has sufficient evidence on the efficacy of HPV vaccination and screening; evidence alone, however, does not deliver services—implementation systems do. Translating this evidence into population-level impact will depend on how well India's programmes address the operational realities of coverage, access, follow-up, quality, trust, and sustainability, with trust functioning, as shown in Figure 1, as the connective mechanism linking awareness to completed care across all five domains.

**Figure 1:**
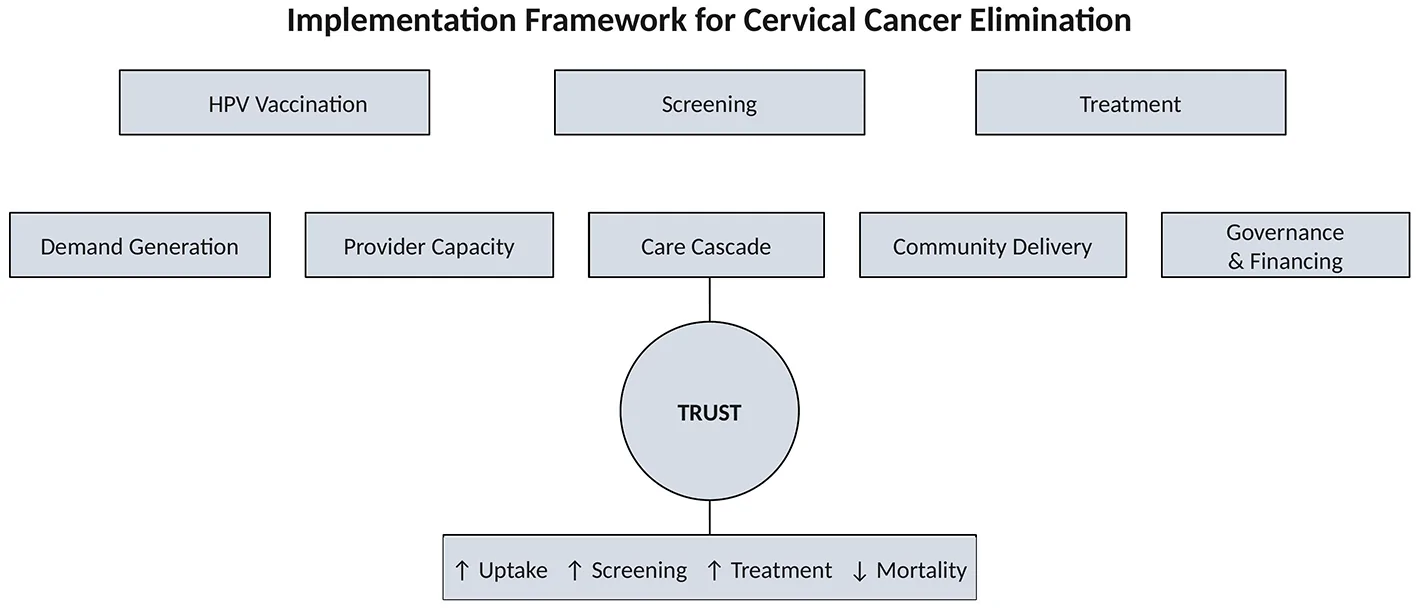
Implementation framework for cervical cancer elimination. This framework illustrates the interdependent domains required for cervical cancer elimination, including HPV vaccination, screening, and treatment, supported by demand generation, provider capacity, care cascade optimization, community-linked service delivery, and governance and financing. Trust functions as a central mechanism linking awareness to uptake and completion of care. Weaknesses at any stage can limit overall program effectiveness.

## Limitations

This synthesis has several limitations. It draws partly on expert presentations and programme experience rather than systematically identified literature. Some cited implementation examples represent emerging or context-specific evidence not yet evaluated using comparable study designs. As a narrative, policy-oriented analysis, it identifies actionable priorities rather than quantifying pooled effects. However, triangulation with peer-reviewed literature and policy sources strengthens the relevance of the findings for policy and practice. The literature cited draws predominantly on global cervical cancer elimination modeling and a limited set of India-specific implementation examples; broader implementation-science, health-systems, and comparative policy literature from other low- and middle-income countries was not systematically reviewed and may be under-represented, and no formal risk-of-bias or quality appraisal was applied to included sources. Because experts were purposively selected rather than sampled systematically, the themes identified may not capture the full range of implementation experience or dissenting views, including documented cases of implementation failure. State- and programme-level examples described in this synthesis, such as Sikkim and the FOGSI/IMA training initiatives, illustrate feasibility in specific contexts rather than national performance, given substantial heterogeneity in health-system capacity across Indian states. Similarly, the reported cost-effectiveness and modeled impact estimates depend on assumptions about sustained coverage, financing, and screening quality, and represent illustrative rather than guaranteed projections of real-world outcomes. Future work would benefit from a more systematic mapping of the implementation-science and health-policy literature, including comparative analyses across low- and middle-income countries.

## Conclusion

Cervical cancer elimination in India is no longer constrained by a lack of effective tools. Vaccines are available, HPV-based screening is increasingly feasible, and implementation experience from India and other LMICs has clarified strategies required for scale. The central challenge is to translate evidence into coordinated action.

India's trajectory is central to global elimination. The challenge is no longer whether prevention at scale is possible, but whether implementation systems can be organized with sufficient urgency, coherence, and accountability to deliver it equitably. Bridging the gap between evidence and delivery will determine whether elimination remains aspirational or becomes an achievable public health milestone within this generation.

## Data Availability

The original contributions presented in the study are included in the article/supplementary material, further inquiries can be directed to the corresponding author.
